# Machine Learning Approaches to Retrieve High-Quality, Clinically Relevant Evidence From the Biomedical Literature: Systematic Review

**DOI:** 10.2196/30401

**Published:** 2021-09-09

**Authors:** Wael Abdelkader, Tamara Navarro, Rick Parrish, Chris Cotoi, Federico Germini, Alfonso Iorio, R Brian Haynes, Cynthia Lokker

**Affiliations:** 1 Health Information Research Unit Department of Health Research Methods, Evidence, and Impact McMaster University Hamilton, ON Canada; 2 Department of Medicine McMaster University Hamilton, ON Canada

**Keywords:** machine learning, bioinformatics, information retrieval, evidence-based medicine, literature databases, systematic review, accuracy, medical literature, clinical support, clinical care

## Abstract

**Background:**

The rapid growth of the biomedical literature makes identifying strong evidence a time-consuming task. Applying machine learning to the process could be a viable solution that limits effort while maintaining accuracy.

**Objective:**

The goal of the research was to summarize the nature and comparative performance of machine learning approaches that have been applied to retrieve high-quality evidence for clinical consideration from the biomedical literature.

**Methods:**

We conducted a systematic review of studies that applied machine learning techniques to identify high-quality clinical articles in the biomedical literature. Multiple databases were searched to July 2020. Extracted data focused on the applied machine learning model, steps in the development of the models, and model performance.

**Results:**

From 3918 retrieved studies, 10 met our inclusion criteria. All followed a supervised machine learning approach and applied, from a limited range of options, a high-quality standard for the training of their model. The results show that machine learning can achieve a sensitivity of 95% while maintaining a high precision of 86%.

**Conclusions:**

Machine learning approaches perform well in retrieving high-quality clinical studies. Performance may improve by applying more sophisticated approaches such as active learning and unsupervised machine learning approaches.

## Introduction

### Background and Significance

Evidence-based medicine (EBM) is identified by three key elements: the best available clinical evidence, clinician expertise, and application of the evidence with consideration of patients’ circumstances, values, and preferences [1]. EBM complements or reduces reliance on expert opinion with a coherent and structured framework for assessing and applying the best evidence to patient care decisions [2]. An obvious and worsening barrier to the implementation of EBM is the continuously growing body of medical literature. According to the National Library of Medicine, over 900,000 new citations were indexed in MEDLINE in 2020, very few of which were relevant to or ready for clinical attention [3]. Searching for the best clinical care evidence is a challenging task for researchers and clinicians, and facilitation of the search process is a necessity [4].

### Search Filters

Search filters, also referred to as hedges, allow researchers, clinicians, and librarians to retrieve evidence from bibliographic databases and journals by filtering searches to return reliable and specific articles to address clinical questions, produce systematic reviews, or inform clinical guidelines [5]. MEDLINE search filters, for example, enable researchers to combine the use of free text with controlled vocabularies like Medical Subject Heading (MeSH) terms and other indexing features to improve search results targeting the clinical question at hand [6,7]. There are search filters that focus on the purpose of a study and its methods or topical content areas [8]. Topical search filters help identify articles based on particular clinical conditions using terms related to that condition [8], while methodological search filters comprise terms that identify articles based on their research purpose [9]. For example, the Hedges project, developed by the Health Information Research Unit at McMaster University, provides search filters for MEDLINE, PsycINFO, and EMBASE using the OVID syntax for a range of purpose categories of articles such as treatment, diagnosis, and prognosis and include methodological terms [4,10,11]. For searches seeking articles on a treatment (purpose), the search hedge includes methodological terms related to clinical or randomized controlled trials (RCTs), while the diagnosis search hedge includes methodological terms including sensitivity and specificity [12].

These search filters were developed to identify high-quality studies based on established critical appraisal criteria for methodological rigor [13-15]. This was done by annotating articles as meeting or not meeting criteria and using the annotated dataset to evaluate the performance of search terms to optimally retrieve the high-quality studies. For RCTs, applying the Cochrane risk for bias tool includes assessing randomization method, allocation concealment, follow-up data for at least 80% of participants, blinding of participants, and outcome assessors [14]. For the Hedges project, the criteria applied to articles by purpose are available online [15].

Clinical search filters are intended to help clinicians, researchers, and policymakers quickly access relevant studies and systematic reviews in a way that can be tailored to the user’s demand [8]. The filters differ in their sensitivity and specificity according to the terms used, databases searched, and precision of the filter [16]. Some filters offer high specificity, which limits the proportion of off-target articles that are retrieved. This is useful for busy clinicians who value the most efficient use of their time in finding relevant evidence quickly. Search filters may also have the option to maximize sensitivity and identify all potentially relevant articles at the cost of including a higher proportion of off-target articles [17], an approach more suited to the conduct of systematic literature reviews.

Although search filters, such as Clinical Queries in PubMed, have been used since 1990 and have continued to work well over the years [18], they have some limitations. One limitation is their partial dependence on MeSH indexing terms, as the process of indexing of articles within MEDLINE can take up to a year for some articles [19]. For diagnostic studies, there is large variability in designs and methods, which may result in largely incomplete literature searches [7]. When applied in the context of conducting a systematic review, the highly specific filters result in missing evidence [7], and the high sensitivity search filters will only partially reduce the time-consuming task of screening retrieved titles and abstracts [20].

### Overview of Machine Learning Applied for Text Processing

Machine learning is a subset of artificial intelligence that refers to a series of computational methods using experience to improve performance or achieve accurate and precise predictions. Experience, in this context, refers to the information made available to the machine for the analysis [21]. A more detailed definition was provided by Mitchell [22]: “A computer program is said to learn from experience (E) with respect to some class of tasks (T) and performance measure (P), if its performance at tasks in T, as measured by P, improves with experience E.”

Machine learning applications have become increasingly popular and essential in health care [23], as the system generates an enormous amount of data every day [24]. Machine learning can identify relevant relations in large health care–generated datasets and derive algorithms that generate accurate predictions [25,26]. For example, machine learning has been used to predict the risk for nosocomial infection by leveraging data from electronic health records [27-29]. A machine learning classifier is a mathematical procedure responsible for identifying the patterns and performing the prediction task on the dataset, while a machine learning model is the output of the algorithm [30]. A machine learning model represents the complete learning process including the training of the algorithm and the used set of features [30].

Another application of machine learning in the health care and biomedical literature is text mining, which refers to the discovery of previously unknown information from unstructured textual data [31]. This is done by converting the text to structured analyzable data using natural language processing (NLP) [32]. With the exponential increase in the amount of information available for clinicians and researchers, both in biomedical literature and electronic health records [33], text mining has been applied for text summarization [34], literature retrieval [35], and evidence grading [36]. Machine learning has also been applied to automate the screening process for systematic reviews, identifying relevant articles while decreasing workload and increasing efficiency [20,37,38]. Semantic analysis, the process of understanding text by interpreting meanings from the unstructured text [39], has been applied to information extraction from the biomedical literature [40].

There are several types of machine learning determined by their mathematical approach [41]. The basic machine learning strategies are supervised learning, unsupervised learning, and reinforced learning [41,42]. Supervised learning relies on a prelabeled training dataset to provide the machine with the necessary input to make accurate predictions [41]. Decision tree (DT), naïve Bayes (NB), and support vector machine (SVM) are common supervised machine learning algorithms [43]. Unsupervised learning does not use labeled data and is mainly used for structuring and organizing data rather than classification [43]. In reinforced learning, the algorithm learns by reacting to its environment and reaches predictions via a reward system [42]. A common machine learning technique is ensemble learning, which combines more than one classifier to perform an individual prediction task. Boosting is one of the commonly used ensemble learners, which combines multiple weak classifiers and converts them into one strong classifier [41]. Neural networks are multilayer mathematical structures consisting of an input layer, an output layer, and a hidden layer (commonly more than one layer) in between [44]. In each layer a series of calculations occurs, leading to better performance [44]. Due to the multilayer nature of neural networks, their field of study is known as deep learning. Neural networks can be supervised, unsupervised, or reinforced [45].

Another appealing application of machine learning approaches to the biomedical literature is to improve retrieval of clinically relevant articles, building on and hopefully overcoming the limitation faced by Boolean searching. Several studies have been conducted to assess the performance of machine learning classifiers to identify specific categories of published articles. For example, Marshall and colleagues [46] applied machine learning to identify RCTs. Del Fiol and colleagues [35] used machine learning to extract only scientifically sound treatment studies from PubMed. However, no systematic review of studies objectively assessing the performance of such machine learning models, ideally comparing their performance to traditional evidence retrieval methods such as validated Boolean search filters or manual critical appraisal by experts in the field, has been performed to date. Such a systematic review would be of critical value in driving future machine learning research aimed at improving the delivery of relevant evidence to the point of care.

### Objective

The objective of this systematic review is to summarize the nature (methods and approaches) and comparative performance (eg, recall and precision) of machine learning approaches that have been applied to retrieve high-quality evidence for clinical consideration from the biomedical literature. High-quality is defined as articles that meet established methodological critical appraisal criteria, with annotated datasets that apply these criteria considered the gold standard.

## Methods

The following subsections describe in detail the steps that were conducted to identify, screen, and abstract data from the included studies.

### Search Strategies

Nine databases were searched from inception to July 8, 2020, to identify relevant articles: Web of Science (title, abstract); MEDLINE; Embase; PsychINFO (title, abstract, keyword, subject terms); Wiley Online Library; ScienceDirect (title, abstract, keyword); CINAHL; IEEE (title, abstract, keywords), and Association of Computer Machinery digital library (title, abstract). The Multidisciplinary Digital Publishing Institute (title, abstract) database was searched on November 17, 2020. The search strategy was developed with a librarian (TN). Search terms related to 4 concepts—machine learning, literature retrieval, high research quality, and biomedical literature—were combined using the AND Boolean operator. The OVID MEDLINE search included the following terms, which were translated for the other databases (mp = multipurpose, searching within the title, original title, abstract, subject heading, name of substance, and registry word fields):

- Machine learning: (neural networks/ or machine learning/ or natural language processing/ or data mining/ or support vector machine/ or (“text categorization” or “text classification” or “text analysis” or “literature mining” or “text mining”).mp)
- Study objective or goal: (“Abstracting and Indexing”/ or “information storage and retrieval”/ or (“article retrieval” or “literature surveillance” or “literature screening” or “article screening” or “evidence search” or “evidence screening” or “evidence review” or “information retrieval” or “literature survey” or “document classification” or “review efficiency” or “citation screening” or “literature databases”).mp.)
- High-quality: (“Sensitivity and Specificity”/ or evidence-based medicine/ or (“quality” or “evidence” or “high-quality” or “clinical trial” or “random*” or “randomized controlled trial” or “sensitivity or specificity” or “accuracy” or “precision”).mp.)

In the Association of Computer Machinery digital library and Multidisciplinary Digital Publishing Institute search queries, terms related to the biomedical literature were included: (“PubMed” or “MEDLINE” or “medical literature” or “Biomedical literature”).

### Study Selection

Articles retrieved by our search queries were collected in a single Research Information Systems file using JabRef software. Deduplication was conducted using both JabRef automatic deduping and Covidence automatic deduplication. We included articles that met the following criteria:

- Reported on the use of a machine learning approach for the retrieval of single studies or systematic reviews concerning the management of health care problems in large biomedical bibliographic databases such as MEDLINE and EMBASE
- Classified retrieved articles based on quality (using a gold standard)
- Used a textual analysis machine learning approach
- Evaluated the performance of the machine learning approach (ie, they present a comparison of retrieval methods or other ways of appraising the performance of the machine learning approach)
- Conducted within the biomedical literature domain
- Published in the English language

### Abstract and Full-Text Screening

Titles and abstracts of all the retrieved articles were screened independently in Covidence.org by two members of the study team. Articles were assessed as relevant, irrelevant, or maybe relevant. The full texts of relevant and maybe relevant articles were then reviewed in duplicate, with conflicts adjudicated by a third team member.

### Data Extraction

A data extraction spreadsheet was developed to gather data regarding the methods of the machine learning approaches as detailed by the survey by Agarwal and Mittal [47] and included details on preprocessing steps, text representation, feature selection, feature extraction, and classifiers used. Additionally, we extracted data specific to the retrieval of high-quality articles such as the quality gold standard, the comparators used to test the machine learning models, and the performance of the developed algorithms.

## Results

### Study Selection

**Figure 1:**
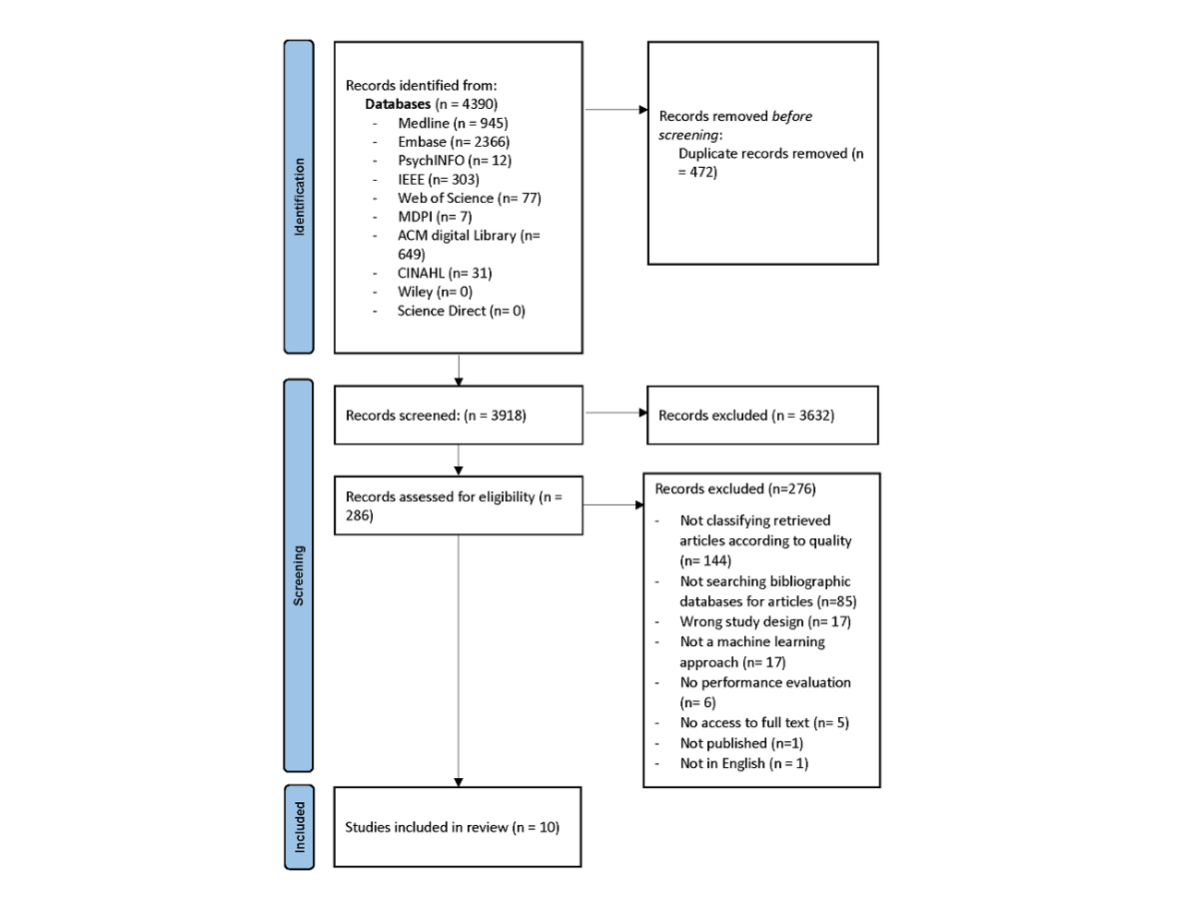
PRISMA flow diagram of the studies identification process for the systematic review [48].

Our search queries retrieved 3918 articles after 472 duplicates were removed; 3632 were excluded during the title and abstract screening for not applying a machine learning approach to biomedical articles. A total of 286 were selected for full-text screening, and 10 articles met our eligibility criteria (Figure 1) [48]. Due to the heterogeneity in the population (retrieved articles), index method (machine learning algorithm used), gold standard, and outcomes (definition of high-quality study), we did not perform a quantitative synthesis of the results.

### Quality Gold Standard

Each study used a quality gold standard database of original studies or systematic reviews that were manually reviewed and annotated by experts based on their scientific soundness and clinical relevance (Table 1). Datasets of articles that met or did not meet standards for quality and relevance were used to train the machine learning models. Four studies used the American College of Physicians (ACP) Journal Club as their quality gold standard [49-52], 3 studies used the Clinical Hedges dataset [4,35,36,53], 2 studies considered articles that were included in treatment clinical guidelines as high quality [54,55], and 1 article used the Cochrane Library as their gold standard [56].

**Table 1:** The quality standard used as the training dataset for developing the classifiers in the included studies.

Author	Quality gold standard
Aphinyanaphongs et al [49]	ACP^a^ Journal Club (treatment class)^b^
Aphinyanaphongs et al [50]	ACP Journal Club (treatment, diagnosis, etiology, prognosis)^b^
Aphinyanaphongs et al [51]	ACP Journal Club (treatment, diagnosis, etiology, prognosis)^b^
Kilicoglu et al [53]	Clinical Hedges^b^
Lin et al [52]	ACP Journal Club (unspecified classes of articles)^b^
Afzal et al [36]	Clinical Hedges^b^
Bian et al [54]	Articles cited in 11 clinical guidelines on the treatment of cardiac, autoimmune, and respiratory diseases
Del Fiol et al [35]	Clinical Hedges^b^
Bian et al [55]	Articles cited in 11 clinical guidelines on the treatment of cardiac, autoimmune, and respiratory diseases
Afzal et al [56]	Cochrane Library Reviews

^a^ACP: American College of Physicians.

^b^Hand searches of articles from approximately 125 clinical journals that were assessed by critical appraisal criteria; articles meeting criteria were then judged by clinicians for clinical relevance. ACP Journal Club includes additional reviews by clinicians.

### Preprocessing Methods

A matrix of the preprocessing steps that were applied to the dataset before developing the classifiers as reported in the included studies is presented in Table 2. Seven of the included studies provided details of their preprocessing steps [35,36,49-51,53,56], which included the conversion of text to lowercase, word-stemming, and removal of stop words. Additionally, 6 studies applied a term weighting method [36,49-51,53,56] to express the importance of a word in each document based on its frequency. Afzal et al [36] used vocabulary pruning by removing off topic-specific frequent terms and rarely occurring terms. Three studies did not specify the steps for their preprocessing steps [52,54,55].

**Table 2:** Preprocessing steps applied to article data for preparing the datasets for machine learning algorithm development.

Author	Text converted to lowercase	Removal of punctuation	Removal of stop words	Porter- stemming	Weighting method	Unique preprocessing considered
Aphinyanaphongs et al [49]	✓^a^	✓	✓	✓	Log frequency with redundancy	NR^b^
Aphinyanaphongs et al [50]	✓	✓	✓	✓	Log frequency with redundancy	NR
Aphinyanaphongs et al [51]	✓	✓	✓	✓	Log frequency with redundancy	Removed infrequent words
Kilicoglu et al [53]	✓	NR	✓	✓	Information gain measure	Removed infrequent words
Lin et al [52]	NR	NR	NR	NR	NR	NR
Afzal et al [36]	✓	NR	✓	✓	TF-IDF^c^	Vocabulary pruning
Bian et al [54]	NR	NR	NR	NR	NR	NR
Del Fiol et al [35]	✓	NR	✓	NR	NR	Removed articles without abstracts, concatenated title, and abstract words
Bian et al [55]	NR	NR	NR	NR	NR	NR
Afzal et al [56]	✓	NR	NR	NR	TF-IDF	Removed articles with missing values

^a^Applied.

^b^NR: not reported.

^c^TF-IDF: term frequency–inverse document frequency.

### Feature Selection

Most of the included articles relied on the text as their features (Multimedia Appendix 1). Seven articles used words from titles and abstracts as their features [35,36,49-51,53,56]. Kilicoglu et al [53] and Afzal et al [36] used article metadata features, Unified Medical Language System features, SemRep semantic prediction, and MeSH terms in combination with the words of titles and abstracts features. Lin et al [52] selected specific features from the citation dataset: journal impact factor, MeSH terms, sample size, *P* value, and confidence intervals. Bian et al [54,55] relied on MEDLINE metadata as well as bibliometric features, which included citation count, journal impact factor, number of comments on PubMed, Altmetric score, study sample size, registration in ClinicalTrials.gov, and article age, and assessed how each feature contributed to the classification. The experiment by Bian et al [55] used only time-agnostic features (features available at the time of an article’s publication), which are journal impact factor, sample size, number of grants, number of authors, number of clinically useful sentences, scientific impact of authors’ institution, numbers of references, page count, registration in ClinicalTrials.gov, and publication in PubMed Central. Afzal et al [56] used automatic feature engineering with RapidMiner software for the title and abstract text feature extraction as part of the multilayer perceptron model.

### Machine Learning Classifier

The majority of the included studies developed multiple algorithms and selected the top-performing one for their main classification tasks (Table 3). Aphinyanaphongs et al [49,50], initially reported their results using SVM, NB, and boosting algorithms in both their 2003 and 2005 experiments; however, they ended up selecting SVM as their top-performing classifier in a separate study [51]. Bian et al [54,55] and Afzal et al [36] compared the performance of multiple classifiers (SVM, NB, DT, k-nearest neighbors, random forest, multilayer perceptron) and selected the best performing for their experiment in the context of the same study (NB, DT, and SVM, respectively). We refer to the classifier that was selected for the classification task as the main classifier.

From the included articles, SVM was the most used classifier. Five studies used an SVM algorithm as one of their main experiment classifiers (Table 3), 2 studies used a neural network as their main classifier; Del Fiol et al [35] used a convolutional neural network (CNN), while Afzal et al [56] used a multilayer feed-forward artificial neural network (ANN). DT algorithms were used in 2 studies for their main text classification function [52,55]. Four of the included studies applied multiple classifying approaches [36,49,50,53].

**Table 3:** Types of machine learning classifiers used in the main experiment to assess performance in each of the included studies.

Author	Naïve Bayes	SVM^a^	Decision tree	Ensemble		Neural network
				Boosting	Stacking	
Aphinyanaphongs et al [49]	✓^b^	✓	N/A^c^	✓	N/A	N/A
Aphinyanaphongs et al [50]	✓	✓	N/A	✓	N/A	N/A
Aphinyanaphongs et al [51]	N/A	✓	N/A	N/A	N/A	N/A
Kilicoglu et al [53]	✓	✓	N/A	✓	✓	N/A
Lin et al [52]	N/A	N/A	✓	N/A	N/A	N/A
Afzal et al [36]	N/A	✓	N/A	N/A	N/A	N/A
Bian et al [54]	✓	N/A	N/A	N/A	N/A	N/A
Del Fiol et al [35]	N/A	N/A	N/A	N/A	N/A	✓
Bian et al [55]	N/A	N/A	✓	N/A	N/A	N/A
Afzal et al [56]	N/A	N/A	N/A	N/A	N/A	✓

^a^SVM: support vector machine.

^b^Applied.

^c^Not applied.

### Comparator for Evaluating the Performance of the Classifiers

As per our inclusion criteria, to evaluate the performance of the machine learning method to classify articles appropriately, articles had to report a comparison of their applied machine learning model to a gold standard method such as gold standard high-quality articles retrieval method, for example, search filters, a manually annotated high-quality articles’ dataset, or a baseline machine learning model for high-quality articles retrieval (Table 4). Aphinyanaphongs et al [49-51] used Clinical Query filters with sensitivity and specificity optimization [57]. The experiment conducted by Kilicoglu et al [53] evaluated their machine learning approach by applying it in a new dataset annotated by experts. The NB high-quality algorithm by Kilicoglu et al [53] was considered a comparator on its own for its high recall and was used as such by Bian and colleagues [54,55], who also used PubMed’s best match as a comparator. Lin et al [52] used accuracy and k-value performance metrics in comparison to the results of the critical appraisal process by experts in the field. Also, Lin et al [52] has applied a comparison between their classifier, which was a DT, to other known text classifiers like SVM and ANN. Afzal et al [36] have used a SVM model for quality articles retrieval and compared its performance to the SVM model proposed by Sarker et al [58], reporting that their classifier achieved a higher performance with their reported features selected.

Del Fiol et al [35] was the first study to incorporate the use of deep learning in quality articles retrieval, relying on a CNN. Del Fiol and colleagues [35] compared their proposed classifier to the PubMed Clinical Queries broad filter since it achieves a nearly perfect recall. Also, they compared their proposed model to McMaster textword search and McMaster balanced search filter created by the Clinical Hedges group to evaluate the capabilities of their model of retrieving recently published evidence and achieving a balance between recall and precision [35]. Afzal et al [36], in their experiment using ANN, compared their model’s results to the CNN results of Del Fiol et al [35], the DT results of Bian et al [55], and their prior experiment using an SVM for quality articles retrieval [35,56]. Also, Afzal et al [56] compared their proposed ANN to well-known algorithms used in the literature like NB, SVM, DT, and gradient boosted trees.

**Table 4:** The gold standard comparator used for evaluating machine learning models in the included studies.

Author	Comparator
Aphinyanaphongs et al [49-51]	PubMed Clinical Query filter [57]
Kilicoglu et al [53]	Testing dataset of 2000 articles annotated by experts (held-out testing dataset to test model’s generalization)
Lin et al [52]	Critical appraisal by domain expert SVM^a^ Artificial neural network
Afzal et al [36]	SVM proposed in Sarker et al [58]
Bian et al [54]	Kilicoglu [53] high-quality classifier PubMed’s relevance sort
Del Fiol et al [35]	PubMed Clinical Query filter McMaster textword search McMaster balanced filter
Bian et al [55]	Kilicoglu et al [53] high-quality classifier PubMed relevance sort High-impact classifier with time-sensitive features included by Bian et al [54]
Afzal et al [56]	Well-known algorithms used in the literature: NB^b^, SVM, DT^c^, GBT^d^ Models from past research by Del Fiol et al [35], Afzal et al [36], and Bian et al [55]

^a^SVM: support vector machine.

^b^NB: naïve Bayes.

^c^DT: decision tree.

^d^GBT: gradient boosted trees.

### Performance Metrics

All included articles applied a supervised machine learning model. Validation by applying a resampling k-fold approach was used in 7 studies. Five used 10-fold cross-validation [35,36,49,52,53], and 2 studies relied on 5-fold cross-validation [50,51]. The most common performance metrics used in the included studies were sensitivity (recall), specificity, accuracy, area under the curve (AUC), F-measure, and precision (Table 5). The recall was generally high, above 85%, across all experiment classifiers except the SVM by Kilicoglu et al [53], and the NB and DT reported by Bian et al [54] and Bian et al [55], respectively, as both had a recall below 30%. Precision ranged from 9% to 86%, with the neural network of Afzal et al [56] and the SVM by Kilicoglu et al [53] the highest. AUC was measured in all studies and ranged from 0.73 to 0.99. Lin et al [52] and Bian et al [54,55] used novel performance metrics in their approaches. In the 2 studies by Bian and colleagues [54,55], performance was primarily determined by calculating the top 20 precision which is the measure of the percentage of true positive citations among the first 20 retrieved citations. Lin et al [52] used Cohen kappa (k-value) as their performance metric, which is the agreement between machine performance (observed value) and gold standard (expected value) [59,60]. Bian et al [54,55] reported a top 20 precision of 34% with their 2017 NB classifier and 24% in their 2019 experiment using a DT classifier. Lin et al [52] reported a k-value of 0.78 in their experiment.

**Table 5:** Highest reported performance characteristics of the main classifier algorithms reported in the included studies.

Classifier and author		Recall^a^		Specificity^b^	Precision^c^		F-score^d^		AUC^e^		Accuracy^f^
**Support vector machine**											
	Aphinyanaphongs et al [49]	0.967	0.87		0.169	0.29^g^		0.98		0.893	
	Aphinyanaphongs et al [50]	0.96	0.86		0.18	0.30^g^		0.97		NR^h^	
	Aphinyanaphongs et al [51]	0.98	0.88		0.305	0.47^g^		0.95		NR	
	Kilicoglu et al [53]	0.229	NR		0.865	0.36		0.96		NR	
	Afzal et al [36]	NR	NR		NR	0.87		0.73		0.785	
**Naïve Bayes**											
	Aphinyanaphongs et al [49]	0.967	0.76		0.091	0.17^g^		0.95		0.787	
	Aphinyanaphongs et al [50]	NR	NR		NR	NR		0.95		NR	
	Kilicoglu et al [53]	0.975	NR		0.138	0.24		0.82		NR	
	Bian et al [54]	0.23	NR		0.33	0.21		NR		NR	
**Boosting**											
	Aphinyanaphongs et al [49]	0.967	0.786		0.099	0.18^g^		0.96		0.804	
	Aphinyanaphongs et al [50]	NR	NR		NR	NR		0.94		NR	
	Kilicoglu et al [53]	0.729	NR		0.823	0.77		0.97		NR	
**Neural network**											
	Del Fiol et al [35]	0.969	NR		0.346	0.51		NR		NR	
	Afzal et al [56]	0.951	NR		0.863	0.9		0.99		0.973	
**Decision tree**											
	Lin et al [52]	NR	NR		NR	NR		NR		0.854	
	Bian et al [55]	0.09	NR		0.39	0.14		NR		NR	
**Stacking**											
	Kilicoglu et al [53]	0.864	NR		0.747	0.801		0.98		NR	

^a^Recall: proportion of correctly identified positives among the real positive.

^b^Specificity: the proportion of actual negatives, which got predicted as the negative (or true negative).

^c^Precision: proportion of correctly identified positives among all classified positives.

^d^F-score: harmonic mean of the precision and recall. F-score is equivalent to F1-score and used interchangeably.

^e^AUC: area under the curve traced out by graphing the true positive rate against the false positive rate. The higher the AUC, the better the classifier prediction.

^f^Accuracy: number of correctly predicted documents out of all classified documents.

^g^Calculated as F-measure=(2*precision*recall)/(precision+recall) using recall and precision when available from the articles.

^h^NR: not reported.

## Discussion

### Summary

To our knowledge, this is the first systematic review of machine learning approaches used to classify scientifically sound and clinically relevant studies from the biomedical literature. All included studies followed a supervised machine learning technique in which the learning algorithm depends on prelabeled data provided for training [41]. Despite the technological advancements from 2003 to 2020 when the studies were published, none reported applying unsupervised or active learning approaches for the classification of articles based on quality. Active learning is a subtype of machine learning in which the learning algorithm is allowed to select the data from which it learns by querying a human operator and can achieve a performance comparable to the standard supervised learning algorithms with fewer labeled data [21]. For example, active learning was used in the recent work by Gates et al [61] and Tsou et al [62], who used Abstrackr, a freely available active machine learning tool that automates the screening of titles and abstracts [63]. Abstrackr achieved 100% sensitivity after screening only 31.8% of the citations in the dataset [63].

There is a limited range of quality standards comprising the prelabeled training datasets across the included articles. ACP Journal Club and the Clinical Hedges follow the same inclusion and exclusion criteria for high-quality evidence [4]. Aphinyanaphongs et al [49] considered an article as high-quality if it were included in ACP Journal Club but considered only those classified as treatment, which limits their results to RCTs. The authors expanded their inclusion to articles tagged as treatment, diagnosis, prognosis, and etiology in their subsequent studies [50,51]. Having consistency across gold standard databases in classifier development strengthens our ability to compare performance. There are, however, limited manually annotated datasets available as these are time consuming and expensive to develop and require consistency and highly skilled people. Using studies that are included in guidelines and systematic reviews, as done by Bian et al [54,55] and Afzal et al [56], leverages screening work that has already been completed to a high standard; however, citations in guidelines may include lower quality evidence in the training process [64].

The limited availability of high-quality dataset options was highlighted by Afzal et al [56], and finding the ideal gold standard training dataset was the most reported limitation in the included studies. In our opinion, the ideal gold standard training dataset should cover some criteria to overcome the limitations reported in the articles. First, the gold standard should be defined by precise criteria for methodological rigor that is created and recognized by experts in the field [50]. Selection criteria for the gold standard should be unbiased. Aphinyanaphongs et al [50] described their concern toward the possibility of a selection bias by the ACP Journal Club editors in a particular year toward a certain topic. Second, the gold standard training dataset should cover a large enough sample of the high-quality class to properly train the model and overcome the class imbalance bias toward the majority class of studies that are not of high quality [63,65]. Third, the gold standard training set should cover multiple health care domains, as Lin et al [52] reported their high-quality dataset was limited only to cardiovascular diseases and would not perform as well if applied to another medical domain. Fourth, the gold standard training dataset should be up to date as much as possible, which was a limitation reported in both studies by Bian et al [54] and Afzal et al [56].

Another possible constraint affecting accurate prediction is the feature selection process. Del Fiol et al [35] stated that using MeSH-based features instead of the sole reliance on text features in their experiment could have improved the precision of their neural network. In consensus with the recommendation of Del Fiol et al [35], some of the included studies provided evidence that the use of a combination of features improves the overall performance of the classifiers. For example, in the experiment by Afzal et al [36], the combination of publication type and MeSH term features in addition to title and abstract features produced the best and the most stable results. Also, Kilicoglu et al [53] proved that the incorporation of MEDLINE citation metadata and Unified Medical Language System features in addition to words of titles and abstracts yielded the best performance. Such important features may not be immediately available at the time of indexing in MEDLINE [19], which poses a challenge in identifying recently published evidence [52,54].

There was a higher rate of incorporating SVM algorithms in the experiments by the study authors. SVMs are known for their high accuracy [66] and their low classification error [41], making them ideal for linear classification. Afzal et al [56] developed an ANN algorithm that had higher accuracy when compared with their previous SVM classifier [36]. Further applications of newer machine learning approaches will advance the knowledge base on these quickly evolving methods. While SVMs currently have good accuracy and low error rates, emerging approaches may well outperform them.

The main purpose of using machine learning in the classification of high-quality articles is to decrease the workload on those performing manual classification without losing relevant articles in the process. Recall, the proportion of correctly identified high-quality articles from the high-quality pool, is the most important metric to be used, followed by precision, the proportion of correctly identified positive articles among all those classified as positive. The included studies reported a range of recall and precision some of which would not meet the objective of identifying the high-quality articles correctly. For example, the NB classifier developed by Bian et al [54] performed significantly less than the NB by Kilicoglu et al [53] and PubMed Best Match in terms of recall (23% vs 55% and 65%, respectively). Despite performing worse in recall, their classifier achieved a higher precision (33% vs 5% and 4%) [54].

Additionally, accuracy, the number of correctly predicted documents out of all classified documents, is considered a common metric for evaluating classifiers; however, its use is considered inappropriate to evaluate imbalanced dataset classification [67]. For example, a classifier labeling all entries as false (given that false is the majority class) would have high accuracy but would fail to perform the needed task of accurately classifying the passing articles (rare class), making it useless [68]. The harmonic mean of the recall and precision measurements is the F-score, and it is used to evaluate the machine learning algorithms implemented on unbalanced datasets [67]. F-score was first used in the study by Kilicoglu et al [53] where the performance of the classifiers was reported using recall, precision, F-score, and AUC, without including accuracy. Additionally, Afzal et al [36] did not rely on recall to compare between multiple classifiers; instead, they used the F-score, precision, and accuracy. Also, they have applied a novel approach to compare between the classifiers, in which they summed the metrics for a classifier with a higher sum reflecting better performance [36].

The highest reported recall in our review was 98% with the SVM developed by Aphinyanaphongs and Aliferis [51], however, the algorithm had low precision of 30.5%. The best balance between recall and precision was achieved by the ANN approach used by Afzal et al [56], which reported a high recall of 95.1% and a high precision of 86.3%, thereby achieving the target of not losing quality literature while decreasing the manual classification workload.

The experiment by Kilicoglu et al [53] assesses the effect of applying 3 different machine learning classifiers (SVM, NB, boosting, and ensemble) trained using the same Clinical Hedges dataset on the overall performance of the resulting models. Using multiple feature set combinations, the highest recall was achieved by the NB classifier, and the highest F-scores were achieved by ensemble (0.80) and text-boosting (0.77) based models [53]. Only the studies by Aphinyanaphongs and colleagues [49,50] and Kilicoglu et al [53] incorporated ensemble techniques in the development of their main classifiers, and their results suggest that using multiple classifiers in combination can improve the balance between recall and precision (the F-score).

### Strengths and Limitations

This is the first systematic review to characterize the machine learning approaches in high-quality article retrieval. When narrowing our research question, we excluded other text summarization and text categorization approaches being used in the biomedical literature. These include but are not limited to studies concerned with the automation of the systematic review process [69,70], biomedical literature summarization [71], and semantic models’ applications in the biomedical literature [72]. Given the technical nature of the application of machine learning approaches for text classification, we expanded our search beyond clinical bibliographic databases to include those which index technical articles.

Across the included studies, some steps were not fully reported in the methods, including preprocessing steps, cross-validation folds, and features selected. To our knowledge, there are no reporting guidelines for machine learning approaches being applied for literature retrieval. The Equator Network includes 6 reporting guidelines for machine learning approaches; however, all 6 are focused on articles applying machine learning in clinical settings [73]. For example, the most recently published guideline focuses on the reporting of interventions involving artificial intelligence in clinical trial protocols [74]. The lack of reporting guidance for the NLP component of machine learning being applied in the biomedical literature creates a noticeable gap in reporting the steps of the applied approach, features used and justification for their use, and inconsistency in the reported performance achieved by the machine. As a result, there was a lack of consistency in the reporting of results and methods provided by the authors, which also limits our ability to compare the performance of the classifiers. Also, one of the limitations developing the review was the inability to directly compare the performance of the models across the included studies because of the different training datasets and the applied settings. Finally, a challenge with machine learning is that the algorithms are considered as being derived in a black box; an enigmatic interpretation that the machines provide findings and predictions without any accompanying explanation [75].

### Conclusion

Despite the longevity of research for the identification of high-quality literature using machine learning, evidence is still scarce and slowly progressing over time, and determining the most reliable approach is difficult as the field is quickly evolving. This slow progression in the field may have been caused by the lack of publicly available standard benchmarks for the identification of high-quality articles biomedical literature to compare the performance of the proposed methods. A similar problem was addressed in the molecular machine learning domain by creating MolecularNet, a large-scale, open-source, and high-quality benchmark for molecular learning algorithms [76]. Our review provides a summary of current approaches and performance of machine learning models applied to retrieve high-quality evidence for clinical consideration from the biomedical literature and highlights the importance of selecting optimal quality gold standard data for training. The findings include that the use of different feature sets in combination with text features is likely to improve the performance of machine learning models. There is a lack of reporting consistency in the literature which makes replication of the experiments difficult. Supervised machine learning has been the focus to date. The rapid development in the field of NLP and the availability of new state of the art techniques such as Bidirectional Encoder Representations from Transformers (BERT) for language understanding [77] and bio-BERT for biomedical text mining [78] hold promise for future advances in the field of information extraction from the biomedical literature. Considering the increasingly available data to apply these approaches to, we anticipate that the performance of classifiers to identify high-quality evidence will continue to grow.

## Appendix

Multimedia Appendix 1 Features selected by the included articles.

## Abbreviations

AUC: area under the receiver operating characteristic curve
ACP: American College of Physicians
ANN: artificial neural network
BERT: Bidirectional Encoder Representations from Transformers
CNN: convolutional neural network
DT: decision tree
EBM: evidence-based medicine
MeSH: Medical Subject Heading
NB: naïve Bayes
NLP: natural language processing
RCT: randomized controlled trial
SVM: support vector machine
