# Supplementary material for: Machine Learning Approaches to Retrieve High-Quality, Clinically Relevant Evidence From the Biomedical Literature: Systematic Review
Source: JMIR Med Inform. 2021 Sep 9;9(9):e30401. doi: 10.2196/30401 (PMC8461527; doi:10.2196/30401)
Supplement: Multimedia Appendix 1 [file medinform_v9i9e30401_app1.pdf]

Supplementary File

Table 1a: Features selected by the included articles

Author (year)	Words of titles and abstracts	MEDLINE citation metadata*	Bibliometric features†	UMLS features‡	Unique features
**Aphinyanaphongs et al. (2003)** [1]	✔	N/A	N/A	N/A	N/A
**Aphinyanaphongs et al. (2005)** [2]	✔	N/A	N/A	N/A	N/A
**Aphinyanaphongs et al. (2006)** [3]	✔	N/A	N/A	N/A	N/A
**Kilicoglu et al. (2009)** [4]	✔	✔	N/A	✔	SemRep semantic predictions
**Lin et al. (2011)** [5]	N/A	N/A	✔	N/A	Sample size, P-value, confidence intervals.
**Afzal et al. (2017)** [6]	✔	✔	N/A	N/A	N/A
**Bian et al. (2017)** [7]	N/A	✔	✔	N/A	Both time-sensitive and time-agnostic features
**Del Fiol et al. (2018)** [8]	✔	N/A	N/A	N/A	N/A
**Bian et al. (2019)** [9]	N/A	✔	✔	N/A	Time-agnostic features only
**Afzal et al. (2020)** [10]	✔	N/A	N/A	N/A	N/A

✔ = applied; N/A = not applied

* MEDLINE citation metadata are the major elements describing a MEDLINE record as indexed by the National Library of Medicine indexers [11]. They include time-sensitive features such as MeSH terms, and publication type, as well as time-agnostic features, for example, registry in ClinicalTrials.gov and authors' affiliations [9].

† Bibliometric features measure the quality of the authors, journals, and institutions [12], such as journal impact factor, and quality of first author's institution [12].

‡ UMLS, Unified Medical Language System, is developed by the National Library of Medicine and it is a collection of biomedical vocabularies which contains biomedical concepts and relations to be used for computer systems development [13].

Table references:

1. Aphinyanaphongs Y, Aliferis CF. Text categorization models for retrieval of high quality articles in internal medicine. AMIA Annu Symp Proc [Internet] United States; 2003;(101209213):31–35. Available from: http://ovidsp.ovid.com/ovidweb.cgi?T=JS&PAGE=reference&D=med5&NEWS=N&AN=14728128
2. Aphinyanaphongs Y, Tsamardinos I, Statnikov A, Hardin D, Aliferis CF. Text categorization models for high-quality article retrieval in internal medicine. J Am Med Inform Assoc [Internet] England; 2005;12(b92, 9430800):207–216. Available from: http://ovidsp.ovid.com/ovidweb.cgi?T=JS&PAGE=reference&D=med6&NEWS=N&AN=15561789
3. Aphinyanaphongs Y, Aliferis C. Prospective validation of text categorization filters for identifying high-quality, content-specific articles in MEDLINE. AMIA Annu Symp Proc [Internet] United States; 2006;(101209213):6–10. Available from: http://ovidsp.ovid.com/ovidweb.cgi?T=JS&PAGE=reference&D=med6&NEWS=N&AN=17238292
4. Kilicoglu H, Demner-Fushman D, Rindflesch TC, Wilczynski NL, Haynes RB. Towards Automatic Recognition of Scientifically Rigorous Clinical Research Evidence. Journal of the American Medical Informatics Association 2009;16(1):25–31. PMID:18952929
5. Lin J-W, Chang C-H, Lin M-W, Ebell MH, Chiang J-H. Automating the process of critical appraisal and assessing the strength of evidence with information extraction technology. J Eval Clin Pract [Internet] England; 2011;17(cwd, 9609066):832–838. Available from: http://ovidsp.ovid.com/ovidweb.cgi?T=JS&PAGE=reference&D=med8&NEWS=N&AN=21707873
6. Afzal M, Hussain M, Haynes RB, Lee S. Context-aware grading of quality evidences for evidence-based decision-making. Health Informatics Journal 2017;25(2):429–445. PMID:28766402
7. Bian J, Morid MA, Jonnalagadda S, Luo G, del Fiol G. Automatic identification of high impact articles in PubMed to support clinical decision making. J Biomed Inform [Internet] United States; 2017;73(100970413, d2m):95–103. Available from: http://ovidsp.ovid.com/ovidweb.cgi?T=JS&PAGE=reference&D=med14&NEWS=N&AN=28756159
8. del Fiol G, Michelson M, Iorio A, Cotoi C, Haynes RB. A Deep Learning Method to Automatically Identify Reports of Scientifically Rigorous Clinical Research from the Biomedical Literature: Comparative Analytic Study. Journal of medical Internet research 2018;20(6):e10281. PMID:29941415
9. Bian J, Abdelrahman S, Shi J, del Fiol G. Automatic identification of recent high impact clinical articles in PubMed to support clinical decision making using time-agnostic features. J Biomed Inform [Internet] United States; 2019;89(100970413, d2m):1–10. Available from: http://ovidsp.ovid.com/ovidweb.cgi?T=JS&PAGE=reference&D=medl&NEWS=N&AN=30468912
10. Afzal M, Park BJ, Hussain M, Lee S. Deep learning based biomedical literature classification using criteria of scientific rigor. Electronics (Switzerland) 2020;9(8):1–12. [doi: 10.3390/electronics9081253]
11. National Library of Medicine. MEDLINE/PubMed Data Element (Field) Descriptions [Internet]. [cited 2021 Nov 6]. Available from: https://www.nlm.nih.gov/bsd/mms/medlineelements.html#ir
12. Fu LD, Aliferis CF. Using content-based and bibliometric features for machine learning models to predict citation counts in the biomedical literature. Scientometrics [Internet] 2010 Oct 3;85(1):257–270. [doi: 10.1007/s11192-010-0160-5]
13. Butte AJ, Chen D. Translational Bioinformatics for Genomic Medicine. Genomic and Personalized Medicine [Internet] Elsevier; 2013. p. 272–286. [doi: 10.1016/B978-0-12-382227-7.00023-9]
